# Pulmonary tuberculosis and diabetes mellitus: Epidemiology, pathogenesis and therapeutic management (Review)

**DOI:** 10.3892/mi.2023.128

**Published:** 2023-12-21

**Authors:** You-Fan Peng

**Affiliations:** Department of Respiratory and Critical Care Medicine, The Affiliated Hospital of Youjiang Medical University for Nationalities, Baise, Guangxi 533000, P.R. China

**Keywords:** pulmonary tuberculosis, diabetes mellitus, epidemiology, pathogenesis, therapeutic management

## Abstract

The dual burden of pulmonary tuberculosis (PTB) and diabetes mellitus (DM) is a major global public health concern. There is increasing evidence to indicate an association between PTB and DM. DM is associated with immune dysfunction and altered immune components. Hyperglycemia weakens the innate immune response by affecting the function of macrophages, dendritic cells, neutrophils, and natural killer cells, and also disrupts the adaptive immune response, thus promoting the susceptibility of PTB in patients with DM. Antituberculosis drugs often cause the impairment of liver and kidney function in patients with PTB, and the infection with *Mycobacterium tuberculosis* weaken pancreatic endocrine function by causing islet cell amyloidosis, which disrupts glucose metabolism and thus increases the risk of developing DM in patients with PTB. The present review discusses the association between PTB and DM from the perspective of epidemiology, pathogenesis, and treatment management. The present review aims to provide information for the rational formulation of treatment strategies for patients with PTB-DM.

## 1. Introduction

Diabetes mellitus (DM) is a metabolic disease with hyperglycemia, which is attributed to the defect of insulin secretion or the impairment of its biological function (1). DM poses a severe threat to human health, and often results in injuries to a number of target organs, such as retinopathy, nephropathy, and peripheral neuropathy (2). As with other organs, the lungs are also a target organ of DM; the lung function of patients with DM has been reported to be markedly decreased, and pulmonary complications have been attested in patients with DM (3,4). Tuberculosis is an ancient disease caused by infection with *Mycobacterium tuberculosis* (5). As a major public health concern, pulmonary tuberculosis (PTB) and DM pose a huge social burden in China (6). It has been reported that PTB-DM-associated comorbidity is high, and that the global prevalence rate of PTB-DM comorbidity is estimated to be 13.73% (7). Although the research progresses of epidemiology, pathogenesis, and treatment management for PTB-DM comorbidity have been summarized, the association between PTB and DM has not been completely described. Therefore, based on recent studies, the aim of the present review was to discuss the epidemiology, pathogenesis, and therapeutic management of patients with PTB-DM.

## 2. Epidemiology

DM leads to an increased risk of developing PTB; an early finding has suggested a link between PTB and DM, and an increased prevalence of DM has been observed in patients with PTB (8). The prevalence of PTB-DM comorbidity is high among the Chinese elderly population (9). A study from Korea found that the prevalence of PTB was significantly higher in patients with DM than in the general population (10); similar results were also demonstrated in Bangladesh (11). In Mexico, the prevalence of DM was shown to have a significant influence on PTB morbidity, and the incidence of PTB was increased by 82.64% in patients with DM, while the incidence of PTB was decreased by 26.77% in patients without DM (12). A recent study suggested a positive association between DM morbidity and PTB in families with tuberculosis (13). Stevenson *et al* (14) found that DM was a significant risk factor for the incidence of PTB, and that the proportion of incident smear-positive tuberculosis was 20.2% due to DM. The prevalence of DM was 18% in patients with PTB, while the prevalence of DM was 8% in patients with suspected PTB (15). In a prospective study, Wang *et al* (16) demonstrated that PTB also increased the risk of developing DM, and that the prevalence of DM in patients with PTB was higher than that in patients without PTB. The prevalence of PTB and DM comorbidity has been summarized in various studies (Table I). These studies suggest a noteworthy association between PTB and DM as regards epidemiology.

## 3. Pathogenesis

**Figure 1:**
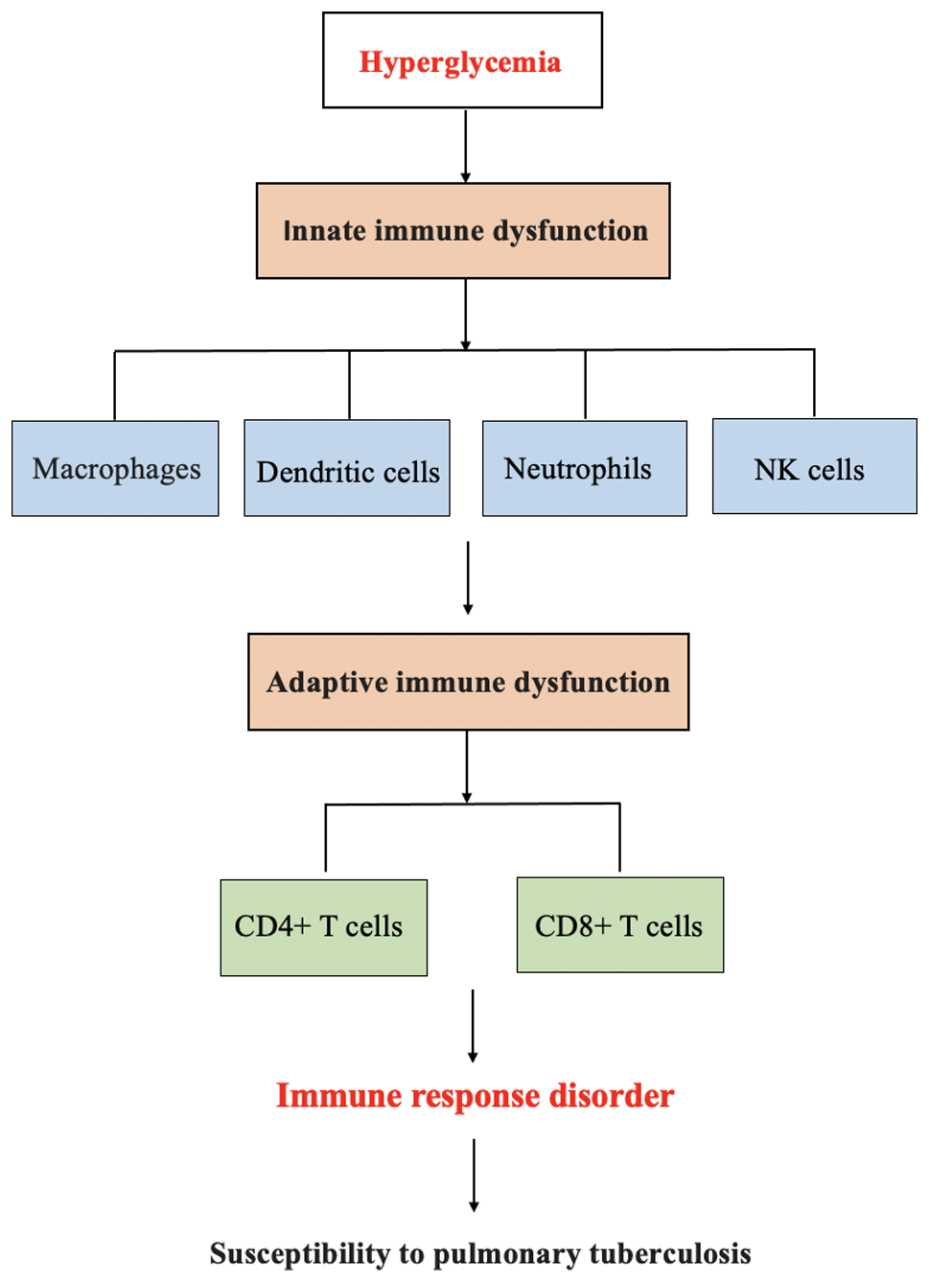
Schematic diagram of the mechanisms through which diabetes mellitus increases the risk of developing pulmonary tuberculosis.

**Figure 2:**
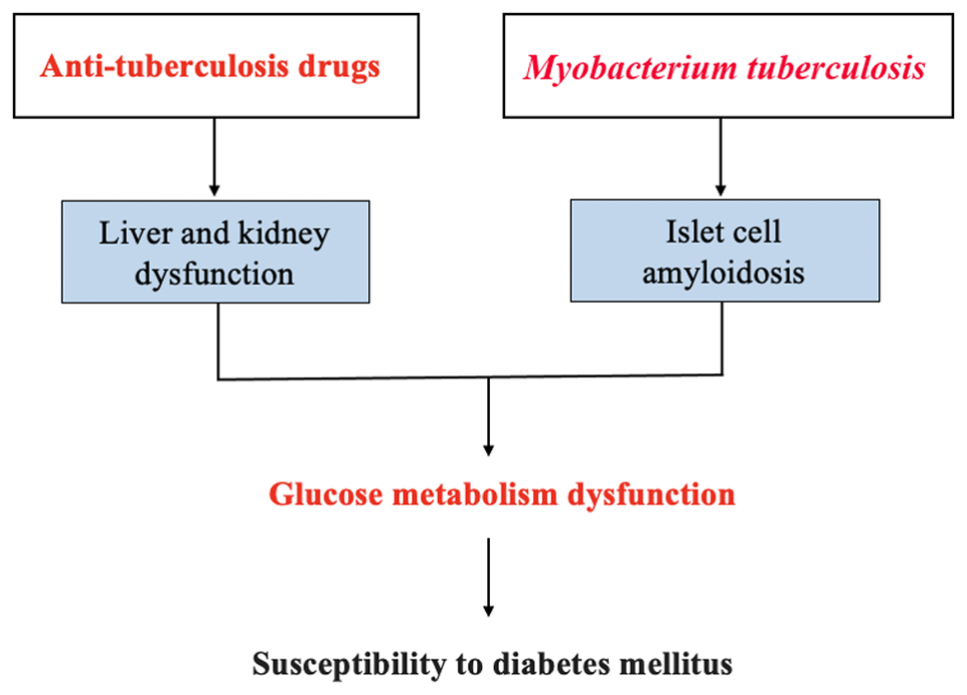
Schematic diagram of the mechanisms through which pulmonary tuberculosis increases the risk of developing diabetes mellitus.

DM increases the risk of PTB by disrupting innate and adaptive immunity responses. In turn, PTB weakens islet cell function by causing islet amyloidosis, and anti-tuberculosis drugs impair blood glucose homeostasis by affecting liver and kidney functions in patients with PTB, which increases the risk of developing DM in patients with PTB. The mechanisms of the interaction between PTB and DM are presented in Figs. 1 and 2.

### Innate immune dysfunction increases the susceptibility to PTB in patients with DM

Macrophages are primary immune cells in the innate immune response of PTB. An increased susceptibility to PTB occurs due to a delayed innate immune response to the alveolar macrophages in *Mycobacterium tuberculosis* infection in DM (17). A recent study found that hyperglycemia affected the phagocytosis ability of macrophages by the change of the expression pattern of recognition receptors in PTB (18). In anti-tuberculosis immunity, the phagocytosis ability of alveolar macrophages is significantly decreased when human alveolar macrophages are directly exposed to hyperglycemia (19). In a previous study on mouse with diet-induced diabetes infected with *Mycobacterium bovis*, the reduced uptake, killing, and production of inflammatory cytokines in alveolar and peritoneal macrophages were observed, compared with macrophages from non-diabetic mice (20). The expression of the macrophage marker, CD14, and its receptors with collagen structure is reduced in the alveolar macrophages of diabetic mice, promoting the susceptibility of diabetic hosts to tuberculosis (21). In a study on diabetic mice with *Mycobacterium fortuitum* infection, the mycobacterium load was found to be significantly increased in the liver, spleen, and lungs compared with controls, and the uptake of mycolic acid coated beads was significantly reduced in macrophages isolated from diabetic mice, indicating the decreased bacterial internalization, killing, and cytokine responses of macrophages isolated from diabetic mice (22). Evidently, these studies suggest that the macrophage-mediated innate immune response is a key pathological mechanism in PTB-DM comorbidity.

Dendritic cells exist in the lung parenchyma, bronchoalveolar fluid, and nasal mucosa of humans, rats, and mice (23). It has been suggested that infection with *Mycobacterium tuberculosis* can induce dendritic cells to mature and migrate into the draining lymph nodes (24,25). The results from a clinical study revealed that the frequency of plasmacytoid and myeloid dendritic cells in PTB-DM comorbidity was decreased at baseline and at 2 months of anti-tuberculosis treatment compared to PTB without DM, and that a significantly increased frequency was observed in plasmacytoid and myeloid dendritic cells when the antituberculosis treatments were successful completed, suggesting that DM may alter the frequency of innate subset distribution of dendritic cells in PTB-DM comorbidity (26). Kumar *et al* (27) found that coincidence DM resulted in a significant reduction in the frequency of plasmacytoid and myeloid dendritic cells in PTB, and that DM altered the distribution of dendritic cells in patients with active and latent tuberculosis.

Neutrophils are another innate cell type that plays a crucial role in the pathogenesis of PTB. Patients with PTB-DM have an elevated peripheral neutrophil count compared to PTB patients without DM (28). Similarly, the neutrophil count of patients with DM infected with *Mycobacterium tuberculosis* is higher than that in patients without DM, and neutrophils exhibit a reduced phagocytic capacity for *Mycobacterium tuberculosis*, suggesting an impaired immune function of neutrophils for the tuberculosis in patients with DM (29). Eruslanov *et al* (30) reported that the accumulation of neutrophils contributed to the development of tuberculosis, indicating that the neutrophils may function as a ‘Trojan horse’ for mycobacterium.

Natural killer (NK) cells are effector cells in an innate immunity response, and play an important role in the host defense against mycobacterial infection (31). Zahran *et al* (32) suggested that the natural killer cell count could be used as a marker to assess disease activity in patients with PTB, and that the peripheral natural killer cell count was a prognostic indicator for patients with active PTB. Of note, the natural killer cells in both peripheral blood and bronchoalveolar lavage have been reported to be significantly increased in patients with PTB-DM compared with PTB patients without DM (33). An increased frequency of tuberculosis antigen-stimulated natural killer cells expressing cytokines has also been observed in PTB-DM (34). Another study demonstrated that natural killer cells promoted the pathological immune response in the infection of *Mycobacterium tuberculosis* in diabetic mice via NK-CD11c^+^ cell interactions (35).

### Adaptive immunity dysfunction increases the susceptibility to PTB in patients with DM

CD4^+^ and CD8^+^ T-cells are critical for *Mycobacterium tuberculosis* in the adaptive immune response (36). The subsets of CD4^+^ T-cells include helper T-cells (Th)1, Th2, Th17, and regulatory T-cells (Tregs) (37). CD4^+^ T-cells are the main antigen-specific cells that are responsible for inhibiting infection with *Mycobacterium tuberculosis* (38). The CD4 cell proportion has been found to be significantly decreased in PTB-DM compared with household contacts (39). Kumar *et al* (40) suggested that PTB-DM increased the frequency of Th1 and Th17 cells, and that DM participated in the altered immune response of PTB. It has been well-documented that the susceptibility of *Mycobacterium tuberculosis* infection is attributed to the defective Th1 cytokine response, while the defective non-specific immune response may increase the susceptibility of *Mycobacterium tuberculosis* in patients with DM (41). Compared with patients with PTB or DM, peripheral blood mononuclear cells exhibit a biased Th1 response to *Mycobacterium tuberculosis* stimulation in patients with PTB-DM (42). It has been shown that in patients with PTB-DM, the imbalance between Treg and effector T-cells is associated with an impaired immune function at pathological sites (43). Increased Th1 and Th17 cytokines exhibit an association with latent tuberculosis (LTB)-pre-diabetes (PDM) and DM comorbidities (44). Following mycobacterial antigen stimulation, the frequency of γδ T-cells expressing Th1 and Th17 is significantly reduced in LTB with DM and/or PDM individuals compared to LTB without DM individuals (45).

Effector CD8^+^ T-cells are cytotoxic cells that produce interferon (IFN)-γ (37). Reportedly, hyperglycemia reduces the counts of CD8^+^ T-lymphocytes in patients with PTB-DM (46). DM modulates the function of CD8^+^ T-cells during LTB infection (47), and it decreases the activity of CD8^+^ T-cells by increasing the differentiation of Th2 and Th17 cells in the regulation of anti-tuberculosis immunity (48). Notably, the frequency of CD4^+^ and CD8^+^ T-cells has been shown to be associated with blood glucose and glycated hemoglobin levels in patients with PTB-DM (49). DM alters the immune responses to tuberculosis, resulting in the induction of CD4 and CD8, which contributes to increased immune pathology in *Mycobacterium tuberculosis* infection (50). The reduced frequency in Th1/Tc1 and Th17/Tc17 cells has been confirmed in LTB-PDM, determining that PDM is also associated with altered immune responses with impaired CD4^+^ and CD8^+^ T-cell functions in LTB (51). CD4 transcription is decreased, while CD8 transcription is increased in patients with PTB-DM, and sputum interleukin (IL)-10 transcription levels are negatively associated with fasting blood glucose and hemoglobin A1c levels in patients with PTB-DM (52). Moreover, the frequency of CD8^+^ T-cells expressing IFN-γ, IL-2, and IL-17F cytokines has been found to be increased following stimulation with mycobacterium antigen in PTB-DM (34).

The IFN-γ response of peripheral blood mononuclear cells is decreased in PTB-DM (53). In diabetic mice infected with *Mycobacterium tuberculosis*, Yamashiro *et al* (54) found the production of IFN-γ was significantly lower than that in non-diabetic mice, and that the decreased number of Th1-related cytokines leaded to an impaired host defense against infection with *Mycobacterium tuberculosis*. In a study performed by Meenakshi *et al* (55), the production of IFN-γ was markedly decreased following *Mycobacterium tuberculosis* stimulation in patients with PTB-DM. Moreover, Stalenhoef *et al* (41) observed decreased levels of non-specific IFN-γ in patients with DM without PTB. However, Gan *et al* (56) suggested that no significant difference was observed in the T-cell IFN-γ responses to *Mycobacterium tuberculosis*-specific antigens between PTB-DM and PTB-non-DM. Thus, further investigations are required to confirm the IFN-γ response in PTB-DM.

### PTB increases the susceptibility to DM

In a previous study, the pancreatic endocrine function was investigated in 51 patients with primary active PTB before and after glucagon stimulation, the results revealed that relative insulin deficiency resulted in persistent hyperglycemia in these patients, and that the delayed concentration peaks of immunoreactive insulin and C peptide were observed in these patients (57). Compared with patients with PTB or DM alone, infection with *Mycobacterium tuberculosis* evidently sustains hyperglycemia in patients with DM (58). The prevention of *Mycobacterium tuberculosis* infection effective reduces weight loss, hyperglycemia, and insulin resistance during DM progression, manifesting that these parameters of glucose metabolism may be affected by *Mycobacterium tuberculosis* (59). Liver and kidney function play indispensable role in maintaining glucose metabolism homeostasis (60,61). Patients with PTB may be subjected to glucose metabolism dysfunction due to the impairment of liver and kidney function during anti-tuberculosis therapy. It has been reported that isoniazid can cause liver damage in anti-tuberculosis treatment (62). Isoniazid, rifampicin, and pyrazinamide cause hepatotoxicity at a probability ranging from 1 to 57% (63). Rifampicin re-administration may lead to an acute kidney injury (64). In addition, the specific amyloidosis of the pancreas is demonstrated in patients with tuberculous infection, while intense islet cell amyloidosis is also commonly observed in DM (65). Of note, islet cell amyloidosis, as a by-product of systemic tubercular infection, is dissolved by rifampicin; thus, it is suggested that infection with *Mycobacterium tuberculosis* may increase the risk of developing DM by causing the islet cell amyloidosis in patients with PTB (65). However, further studies are warranted to elucidate the mechanisms through which PTB increases the risk of developing DM.

## 4. Therapeutic management of patients with PTB-DM

### Glucose control and PTB outcomes

DM is associated with the prognosis in patients with PTB, and DM has an adverse effect on the outcomes of PTB treatments (66). In patients newly diagnosed with PTB with DM or PDM, DM and hyperglycemia have been found to be associated with an increased bacterial burden of *Mycobacterium tuberculosis* and the risk of PTB transmission (15), and a poor blood glucose control has been demonstrated to increase the risk of PTB in patients with DM (67,68). Gil-Santana *et al* (69) suggested that DM was associated with the severity of PTB. An association between blood glucose levels and the computed tomography severity score has been found in patients with PTB-DM (70). Recently, a prospective cohort study indicated that patients with PTB with DM had a poorer prognosis than those without DM (71). A poor blood glucose control has been shown to be associated with the outcomes of PTB treatments, while improved hyperglycemia can reduce the effects of DM in patients with PTB (72). A meta-analysis suggested that DM was associated with an increased risk of a poor treatment response in patients with PTB, and that DM might increase the risk of PTB resistance (73). PTB patients with DM are more likely to have a failed response to anti-tuberculosis therapy than those without DM (12). In addition, DM is a risk factor for the positive rate of tuberculosis culture after receiving anti-tuberculosis treatment, anti-tuberculosis treatment failure, and mortality in patients with PTB (74). Evidently, blood glucose control is associated with the outcomes of anti-tuberculosis treatment in patients with PTB-DM.

### DM and anti-tuberculosis treatment

Anti-tuberculosis medications are divided into first- and second-line drugs; first-line anti-tuberculosis drugs include rifampicin, isoniazid, pyrazine, ethambutol, and streptomycin; these have a high efficiency and acceptable toxicity; second-line anti-tuberculosis drugs, such as fluoroquinolones, aminoglycosides, are used for multidrug resistance (75-77). *Mycobacterium tuberculosis* has the ability to manipulate innate and adaptive immune responses, which is known as the tuberculous escape mechanism; thus, *Mycobacterium tuberculosis* can avoid the intracellular killing and macrophage phagocytosis by the escape mechanism; however, hyperglycemia aggravates its escape mechanism, leading to an increased risk of anti-tuberculosis treatment failure in patients with PTB-DM (78). Poor blood glucose control increases the risk of pyrazinamide treatment failure, and DM also appears to influence the pharmacokinetic-pharmacodynamic association between isoniazid and rifampicin (79). Babalik *et al* (80) demonstrated that the plasma concentrations of isoniazid and rifampicin were reduced by ~50% in patients with PTB-DM; their data indicate that hyperglycemia significantly reduces the efficiency of anti-tuberculosis drugs.

DM significantly increases the risk of resistance to anti-tuberculosis medications (81). The use of hypoglycemic drugs significantly increases the effectiveness of anti-tuberculosis treatment in patients with PTB-DM. For instance, metformin can be used as an effective auxiliary anti-tuberculosis drug in patients with PTB-DM, as it can improve sputum culture transformation following 2 months of therapy (82); secondly, metformin use has been reported to be significantly associated with reduced mortality during antituberculosis treatment in patients with PTB-DM (83). Therefore, clinically, additional attention needs to be paid to the treatment and management of patients with PTB-DM; a satisfactory blood glucose control increases the efficacy of anti-tuberculosis treatment in these patients.

## 5. Conclusions and future perspectives

The present review provided an update on PTB-DM from the perspectives of epidemiology, pathogenesis, and treatment; however, there are still a number of issues that need to be further addressed in the future. First, the majority of epidemiological studies are based on a cross-sectional design; thus, further prospective cohort studies with large sample sizes are required to clarify a causality between PTB and DM. Second, islet cell dysfunction plays a key role in the pathogenesis of DM; current studies mainly focus on the effects of hyperglycemia on the susceptibility to *Mycobacterium tuberculosis*; however, few studies have explored the mechanisms through which *Mycobacterium tuberculosis* infection affects islet cell function in patients with PTB-DM.

In conclusion, the present review provides insight for future studies on the link between PTB and DM, particularly as regards the mechanisms of their interaction. A better understanding of the mechanisms of the association between DM and PTB may help to formulate effective treatment strategies with which to reduce the double burden of PTB-DM.

## Figures and Tables

**Table I:** Prevalence of pulmonary tuberculosis and diabetes mellitus comorbidity in different studies.

Authors, year of publication	Study design	Study population	Main results	(Refs.)
Mugusi *et al*, 1990	Cross-sectional	506 Patients with PTB, and 693 individuals of an urban community	A crude DM prevalence rate was 4.0% in patients with PTB; prevalence rate of DM was 0.9% in individuals of an urban community	(8)
Wu *et al*, 2022	Cross-sectional	784 Patients with PTB	8.12% Patients with PTB-DM	(9)
Lee *et al*, 2017	Cross-sectional	1,044 Patients with active PTB, and 14,655 contemporaneous general individuals	The prevalence of DM was 24.2% in patients with PTB and 11.6% in contemporaneous general individuals	(10)
Rahim *et al*, 2012	Prospective	17,344 Patients with DM	The prevalence of patients with PTB was 213.3/100,000 in patients with DM; an estimated incidence of PTB was 101/100,000 patients in Bangladesh	(11)
Delgado-Sánchez *et al*, 2015	Cross-sectional	181,378 Patients with PTB	19.29% Patients were diagnosed with DM, during the study period, the incidence rate of PTB with DM increased by 82.64% while PTB without DM decreased by 26.77%	(12)
Guo *et al*, 2022	Cross-sectional	801 Patients with PTB, and 972 household contacts	The prevalence of DM in culture (+) PTB patients 9.2% was higher than the culture (-) PTB patients 7.8% and the non-PTB 3.4% subjects	(13)
Stevenson *et al*, 2007	Cross-sectional	139,000 Patients with PTB	DM accounted for 14.8% in patients with PTB; the proportion of incident smear-positive tuberculosis was 20.2% due to DM	(14)
Mave *et al*, 2017	Cross-sectional	890 Patients with PTB, and 552 patients with suspected PTB	The prevalence of DM was 18% in patients with PTB; DM was 8% in patients with suspected PTB	(15)
Wang *et al*, 2013	Prospective study	6,382 Patients with PTB, and 6,674 non-PTB patients	The prevalence of DM in patients with PTB 6.3% was significantly higher than non-TB patients 4.7%	(16)

## Data Availability

Not applicable.

## References

[2] Zheng Y, Ley SH, Hu FB (2018). Global aetiology and epidemiology of type 2 diabetes mellitus and its complications. Nat Rev Endocrinol.

[3] Kaparianos A, Argyropoulou E, Sampsonas F, Karkoulias K, Tsiamita M, Spiropoulos K (2008). Pulmonary complications in diabetes mellitus. Chron Respir Dis.

[4] Ali MO (2014). Pulmonary complications in diabetes mellitus. Mymensingh Med J.

[5] Natarajan A, Beena PM, Devnikar AV, Mali S (2020). A systemic review on tuberculosis. Indian J Tuberc.

[6] Zheng C, Hu M, Gao F (2017). Diabetes and pulmonary tuberculosis: A global overview with special focus on the situation in Asian countries with high TB-DM burden. Glob Health Action.

[7] Li M, Chen T, Hua Z, Yan H, Wang D, Li Z, Kang Y, Zhu N, Li C (2021). Global, regional, and national prevalence of diabetes mellitus in patients with pulmonary tuberculosis: A systematic review and meta-analysis. Diabetol Metab Syndr.

[8] Mugusi F, Swai AB, Alberti KG, McLarty DG (1990). Increased prevalence of diabetes mellitus in patients with pulmonary tuberculosis in Tanzania. Tubercle.

[9] Wu Q, Wang M, Zhang Y, Wang W, Ye TF, Liu K, Chen SH (2022). Epidemiological characteristics and their influencing factors among pulmonary tuberculosis patients with and without diabetes mellitus: A survey study from drug resistance surveillance in east china. Front Public Health.

[10] Lee EH, Lee JM, Kang YA, Leem AY, Kim EY, Jung JY, Park MS, Kim YS, Kim SK, Chang J, Kim SY (2017). Prevalence and impact of diabetes mellitus among patients with active pulmonary tuberculosis in South Korea. Lung.

[11] Rahim Z, Momi MS, Saha SK, Zaman K, Uddin KN, Jamil SN, Nahar N, Khan AK, Cooreman EA, Ahmed M (2012). Pulmonary tuberculosis in patients with diabetes mellitus in Bangladesh. Int J Tuberc Lung Dis.

[12] Delgado-Sánchez G, García-García L, Castellanos-Joya M, Cruz-Hervert P, Ferreyra-Reyes L, Ferreira-Guerrero E, Hernández A, Ortega-Baeza VM, Montero-Campos R, Sulca JA (2015). Association of pulmonary tuberculosis and diabetes in Mexico: Analysis of the national tuberculosis registry 2000-2012. PLoS One.

[13] Guo S, Lei S, Li J, Li L, Chen H, Chongsuvivatwong V (2022). Gradient association between pulmonary tuberculosis and diabetes mellitus among households with a tuberculosis case: A contact tracing-based study. Sci Rep.

[14] Stevenson CR, Forouhi NG, Roglic G, Williams BG, Lauer JA, Dye C, Unwin N (2007). Diabetes and tuberculosis: The impact of the diabetes epidemic on tuberculosis incidence. BMC Public Health.

[15] Mave V, Meshram S, Lokhande R, Kadam D, Dharmshale S, Bharadwaj R, Kagal A, Pradhan N, Deshmukh S, Atre S (2017). Prevalence of dysglycemia and clinical presentation of pulmonary tuberculosis in Western India. Int J Tuberc Lung Dis.

[16] Wang Q, Ma A, Han X, Zhao S, Cai J, Ma Y, Zhao J, Wang Y, Dong H, Zhao Z (2013). Prevalence of type 2 diabetes among newly detected pulmonary tuberculosis patients in China: A community based cohort study. PLoS One.

[17] Vallerskog T, Martens GW, Kornfeld H (2010). Diabetic mice display a delayed adaptive immune response to Mycobacterium tuberculosis. J Immunol.

[18] Panda S, Seelan DM, Faisal S, Arora A, Luthra K, Palanichamy JK, Mohan A, Vikram NK, Gupta NK, Ramakrishnan L, Singh A (2022). Chronic hyperglycemia drives alterations in macrophage effector function in pulmonary tuberculosis. Eur J Immunol.

[19] Vance J, Santos A, Sadofsky L, Morice A, Cervantes J (2019). Effect of high glucose on human alveolar macrophage phenotype and phagocytosis of mycobacteria. Lung.

[20] Alim MA, Sikder S, Sathkumara H, Kupz A, Rush CM, Govan BL, Ketheesan N (2019). Dysregulation of key cytokines may contribute to increased susceptibility of diabetic mice to Mycobacterium bovis BCG infection. Tuberculosis (Edinb).

[21] Martinez N, Ketheesan N, West K, Vallerskog T, Kornfeld H (2016). Impaired recognition of mycobacterium tuberculosis by alveolar macrophages from diabetic mice. J Infect Dis.

[22] Alim MA, Sikder S, Bridson TL, Rush CM, Govan BL, Ketheesan N (2017). Anti-mycobacterial function of macrophages is impaired in a diet induced model of type 2 diabetes. Tuberculosis (Edinb).

[23] Sertl K, Takemura T, Tschachler E, Ferrans VJ, Kaliner MA, Shevach EM (1986). Dendritic cells with antigen-presenting capability reside in airway epithelium, lung parenchyma, and visceral pleura. J Exp Med.

[24] McWilliam AS, Marsh AM, Holt PG (1997). Inflammatory infiltration of the upper airway epithelium during Sendai virus infection: Involvement of epithelial dendritic cells. J Virol.

[25] Holt PG, Stumbles PA, McWilliam AS (1999). Functional studies on dendritic cells in the respiratory tract and related mucosal tissues. J Leukoc Biol.

[26] Kumar NP, Moideen K, Sivakumar S, Menon PA, Viswanathan V, Kornfeld H, Babu S (2016). Modulation of dendritic cell and monocyte subsets in tuberculosis-diabetes co-morbidity upon standard tuberculosis treatment. Tuberculosis (Edinb).

[27] Kumar NP, Moideen K, Dhakshinraj SD, Banurekha VV, Nair D, Dolla C, Kumaran P, Babu S (2015). Profiling leucocyte subsets in tuberculosis-diabetes co-morbidity. Immunology.

[28] Andrade BB, Kumar NP, Sridhar R, Banurekha VV, Jawahar MS, Nutman TB, Sher A, Babu S (2014). Heightened plasma levels of heme oxygenase-1 and tissue inhibitor of metalloproteinase-4 as well as elevated peripheral neutrophil counts are associated with TB-diabetes comorbidity. Chest.

[29] Raposo-García S, Guerra-Laso JM, García-García S, Juan-García J, López-Fidalgo E, Diez-Tascón C, Nebreda-Mayoral T, López-Medrano R, Rivero-Lezcano OM (2017). Immunological response to Mycobacterium tuberculosis infection in blood from type 2 diabetes patients. Immunol Lett.

[30] Eruslanov EB, Lyadova IV, Kondratieva TK, Majorov KB, Scheglov IV, Orlova MO, Apt AS (2005). Neutrophil responses to Mycobacterium tuberculosis infection in genetically susceptible and resistant mice. Infect Immun.

[31] Vankayalapati R, Barnes PF (2009). Innate and adaptive immune responses to human Mycobacterium tuberculosis infection. Tuberculosis.

[32] Zahran WA, Ghonaim MM, Koura BA, El-Banna H, Ali SM, El-Sheikh N (2006). Human natural killer T cells (NKT), NK and T cells in pulmonary tuberculosis: Potential indicators for disease activity and prognosis. Egypt J Immunol.

[33] Zhang Q, Xiao HP, Cui HY, Sugawara I (2011). Significant increase in natural-killer T cells in patients with tuberculosis complicated by type 2 diabetes mellitus. J Int Med Res.

[34] Kumar NP, Sridhar R, Nair D, Banurekha VV, Nutman TB, Babu S (2015). Type 2 diabetes mellitus is associated with altered CD8(+) T and natural killer cell function in pulmonary tuberculosis. Immunology.

[35] Cheekatla SS, Tripathi D, Venkatasubramanian S, Nathella PK, Paidipally P, Ishibashi M, Welch E, Tvinnereim AR, Ikebe M, Valluri VL (2016). NK-CD11c+ cell crosstalk in diabetes enhances IL-6-mediated inflammation during mycobacterium tuberculosis infection. PLoS Pathog.

[36] Prezzemolo T, Guggino G, La Manna MP, Di Liberto D, Dieli F, Caccamo N (2014). Functional signatures of human CD4 and CD8 T cell responses to mycobacterium tuberculosis. Front Immunol.

[37] St Paul M, Ohashi PS (2020). The roles of CD8^+^ T cell subsets in antitumor immunity. Trends Cell Biol.

[38] Mayer-Barber KD, Barber DL (2015). Innate and adaptive cellular immune responses to mycobacterium tuberculosis infection. Cold Spring Harb Perspect Med.

[39] Ponnana M, Pydi S, Gaddam S (2020). Enumeration of lymphocyte subsets during follow-up in the pulmonary tuberculosis patients with co morbid diabetes mellitus. Clin Chim Acta.

[40] Kumar NP, Sridhar R, Banurekha VV, Jawahar MS, Nutman TB, Babu S (2013). Expansion of pathogen-specific T-helper 1 and T-helper 17 cells in pulmonary tuberculosis with coincident type 2 diabetes mellitus. J Infect Dis.

[41] Stalenhoef JE, Alisjahbana B, Nelwan EJ, van der Ven-Jongekrijg J, Ottenhoff TH, van der Meer JW, Nelwan RH, Netea MG, van Crevel R (2008). The role of interferon-gamma in the increased tuberculosis risk in type 2 diabetes mellitus. Eur J Clin Microbiol Infect Dis.

[42] Fernández RDV, Díaz A, Bongiovanni B, Gallucci G, Bértola D, Gardeñez W, Lioi S, Bertolin Y, Galliano R, Bay ML (2020). Evidence for a more disrupted immune-endocrine relation and cortisol immunologic influences in the context of tuberculosis and type 2 diabetes comorbidity. Front Endocrinol (Lausanne).

[43] Sun Q, Zhang Q, Xiao H, Cui H, Su B (2012). Significance of the frequency of CD4+CD25+CD127- T-cells in patients with pulmonary tuberculosis and diabetes mellitus. Respirology.

[44] Kathamuthu GR, Kumar NP, Moideen K, Dolla C, Kumaran P, Babu S (2022). Multi-dimensionality immunophenotyping analyses of MAIT cells expressing Th1/Th17 cytokines and cytotoxic markers in latent tuberculosis diabetes comorbidity. Pathogens.

[45] Kathamuthu GR, Kumar NP, Moideen K, Menon PA, Babu S (2021). Decreased frequencies of Gamma/Delta T cells expressing Th1/Th17 cytokine, cytotoxic, and immune markers in latent tuberculosis-diabetes/pre-diabetes comorbidity. Front Cell Infect Microbiol.

[46] Wei R, Li P, Xue Y, Liu Y, Gong W, Zhao W (2022). Impact of diabetes mellitus on the immunity of tuberculosis patients: A retrospective, cross-sectional study. Risk Manag Healthc Policy.

[47] Kumar NP, Moideen K, George PJ, Dolla C, Kumaran P, Babu S (2016). Impaired cytokine but enhanced cytotoxic marker expression in mycobacterium tuberculosis-induced CD8+ T cells in individuals with type 2 diabetes and latent mycobacterium tuberculosis infection. J Infect Dis.

[48] Wang X, Ma A, Han X, Chan L, Liang H, Litifu A, Xue F (2018). T cell profile was altered in pulmonary tuberculosis patients with type 2 diabetes. Med Sci Monit.

[49] Kumar NP, Moideen K, Viswanathan V, Kornfeld H, Babu S (2016). Effect of standard tuberculosis treatment on naive, memory and regulatory T-cell homeostasis in tuberculosis-diabetes co-morbidity. Immunology.

[50] Kumar S, Lakhiwal R, Singh CP, Bhandiwad C, Sharma N, Singhal V, Chakranarayan A (2022). Study of correlation of CD4, CD8 count with tuberculous pneumonia and non tuberculous bacterial pneumonia in type 2 diabetes mellitu. J Assoc Physicians India.

[51] Kumar NP, Moideen K, Dolla C, Kumaran P, Babu S (2017). Prediabetes is associated with the modulation of antigen-specific Th1/Tc1 and Th17/Tc17 responses in latent Mycobacterium tuberculosis infection. PLoS One.

[52] Mily A, Sarker P, Taznin I, Hossain D, Haq MA, Kamal SMM, Agerberth B, Brighenti S, Raqib R (2020). Slow radiological improvement and persistent low-grade inflammation after chemotherapy in tuberculosis patients with type 2 diabetes. BMC Infect Dis.

[53] Guo Q, Zhang J, Li G, Liu S, Xiao G, Bi J, Li F, Zhang S, Ou M, He X (2020). Elevated antigen-specific IFN-γ responses in bronchoalveolar lavage fluid impervious to clinical comorbidities improve the pulmonary tuberculosis diagnosis. Tuberculosis (Edinb).

[54] Yamashiro S, Kawakami K, Uezu K, Kinjo T, Miyagi K, Nakamura K, Saito A (2005). Lower expression of Th1-related cytokines and inducible nitric oxide synthase in mice with streptozotocin-induced diabetes mellitus infected with Mycobacterium tuberculosis. Clin Exp Immunol.

[55] Meenakshi P, Ramya S, Lavanya J, Vijayalakshmi V, Sumanlatha G (2016). Effect of IFN-γ, IL-12 and IL-10 cytokine production and mRNA expression in tuberculosis patients with diabetes mellitus and their household contacts. Cytokine.

[56] Gan SH, KhinMar KW, Barkham TM, Koh CK, Shen L, Wang YT, Chee CB (2014). Interferon-γ responses to Mycobacterium tuberculosis-specific antigens in diabetes mellitus. Eur Respir J.

[57] Karachunskiĭ MA, Balabolkin MI, Beglarian NR (1995). Changes in carbohydrate metabolism in patients with tuberculosis. Vestn Ross Akad Med Nauk.

[58] Chen H, Su L, Bao J, Zhang K, Li Y, Mao E (2022). The impact of pulmonary tuberculosis on immunological and metabolic features of diabetic patients. Front Immunol.

[59] Segura-Cerda CA, Marquina-Castillo B, Lozano-Ordaz V, Mata-Espinosa D, Barrios-Payán JA, López-Torres MO, Aceves-Sánchez MJ, Bielefeldt-Ohmann H, Hernández-Pando R, Flores-Valdez MA (2020). BCG and BCGΔBCG1419c protect type 2 diabetic mice against tuberculosis via different participation of T and B lymphocytes, dendritic cells and pro-inflammatory cytokines. NPJ Vaccines.

[60] Adeva-Andany MM, Pérez-Felpete N, Fernández-Fernández C, Donapetry-García C, Pazos-García C (2016). Liver glucose metabolism in humans. Biosci Rep.

[61] Legouis D, Faivre A, Cippà PE, de Seigneux S (2022). Renal gluconeogenesis: An underestimated role of the kidney in systemic glucose metabolism. Nephrol Dial Transplant.

[62] Gray EL, Goldberg HF (2016). Baseline abnormal liver function tests are more important than age in the development of isoniazid-induced hepatoxicity for patients receiving preventive therapy for latent tuberculosis infection. Intern Med J.

[63] Becker MW, Schwambach KH, Lunardelli M, Blatt CR (2021). Overview of drug induced liver injury in Brazil: What is the role of public health policy on the evidence?. World J Gastrointest Pharmacol Ther.

[64] Covic A, Golea O, Segall L, Meadipudi S, Munteanu L, Nicolicioiu M, Tudorache V, Covic M, Goldsmith DJ (2004). A clinical description of rifampicin-induced acute renal failure in 170 consecutive cases. J Indian Med Assoc.

[65] Broxmeyer L (2005). Diabetes mellitus, tuberculosis and the mycobacteria: Two millenia of enigma. Med Hypotheses.

[66] Sahakyan S, Petrosyan V, Abrahamyan L (2020). Diabetes mellitus and treatment outcomes of pulmonary tuberculosis: A cohort study. Int J Public Health.

[67] Ahmed M, Omer I, Osman SM, Ahmed-Abakur EH (2017). Association between pulmonary tuberculosis and Type 2 diabetes in Sudanese patients. Int J Mycobacteriol.

[68] Wang Y, Dou M, Kou T, Liu Y, Lv W, Han L, Wang N, Ma A, Kok FJ, Schouten EG, Wang Q (2021). Risk of having pulmonary tuberculosis in type 2 diabetes: A hospital-based matched case-control study. Asia Pac J Clin Nutr.

[69] Gil-Santana L, Almeida-Junior JL, Oliveira CA, Hickson LS, Daltro C, Castro S, Kornfeld H, Netto EM, Andrade BB (2016). Diabetes is associated with worse clinical presentation in tuberculosis patients from Brazil: A retrospective cohort study. PLoS One.

[70] Ren Y, Ren H, Tian Q, Li X, Liu Y (2022). The relationship between computed tomography appearance of pulmonary tuberculosis and blood glucose levels in 763 diabetes mellitus patients with pulmonary tuberculosis: A comparative study. Endocrine.

[71] Buasroung P, Petnak T, Liwtanakitpipat P, Kiertiburanakul S (2022). Prevalence of diabetes mellitus in patients with tuberculosis: A prospective cohort study. Int J Infect Dis.

[72] Chiang CY, Bai KJ, Lin HH, Chien ST, Lee JJ, Enarson DA, Lee TI, Yu MC (2015). The influence of diabetes, glycemic control, and diabetes-related comorbidities on pulmonary tuberculosis. PLoS One.

[73] Huangfu P, Ugarte-Gil C, Golub J, Pearson F, Critchley J (2019). The effects of diabetes on tuberculosis treatment outcomes: An updated systematic review and meta-analysis. Int J Tuberc Lung Dis.

[74] Ma Y, Huang ML, Li T, DU J, Shu W, Xie SH, Wang HH, Zhu GF, Tan SY, Fu YY (2017). Role of diabetes mellitus on treatment effects in drug-susceptible initial pulmonary tuberculosis patients in China. Biomed Environ Sci.

[75] Brunton L, Chapner B, Knollmann B

[76] Katzung BG, Mastres SB, Trevor AJ

[77] Parida SK, Axelsson-Robertson R, Rao MV, Singh N, Master I, Lutckii A, Keshavjee S, Andersson J, Zumla A, Maeurer M (2015). Totally drug- resistant tuberculosis and adjunct therapies. J Intern Med.

[78] Novita BD (2019). Metformin: A review of its potential as enhancer for anti tuberculosis efficacy in diabetes mellitus-tuberculosis coinfection patients. Indian J Tuberc.

[79] Alfarisi O, Mave V, Gaikwad S, Sahasrabudhe T, Ramachandran G, Kumar H, Gupte N, Kulkarni V, Deshmukh S, Atre S (2018). Effect of diabetes mellitus on the pharmacokinetics and pharmacodynamics of tuberculosis treatment. Antimicrob Agents Chemother.

[80] Babalik A, Ulus IH, Bakirci N, Kuyucu T, Arpag H, Dagyildizi L, Capaner E (2013). Plasma concentrations of isoniazid and rifampin are decreased in adult pulmonary tuberculosis patients with diabetes mellitus. Antimicrob Agents Chemother.

[81] Hsu AH, Lee JJ, Chiang CY, Li YH, Chen LK, Lin CB (2013). Diabetes is associated with drug-resistant tuberculosis in Eastern Taiwan. Int J Tuberc Lung Dis.

[82] Lee YJ, Han SK, Park JH, Lee JK, Kim DK, Chung HS, Heo EY (2018). The effect of metformin on culture conversion in tuberculosis patients with diabetes mellitus. Korean J Intern Med.

[83] Degner NR, Wang JY, Golub JE, Karakousis PC (2018). Metformin use reverses the increased mortality associated with diabetes mellitus during tuberculosis treatment. Clin Infect Dis.

